# Immunological Basis of Genesis of Hepatocellular Carcinoma: Unique Challenges and Potential Opportunities through Immunomodulation

**DOI:** 10.3390/vaccines8020247

**Published:** 2020-05-23

**Authors:** Kumar Jayant, Nagy Habib, Kai W. Huang, Mauro Podda, Jane Warwick, Ramesh Arasaradnam

**Affiliations:** 1 Warwick Medical School, University of Warwick, Coventry CV4 7H, UK; j.warwick@warwick.ac.uk (J.W.); R.Arasaradnam@warwick.ac.uk (R.A.); 2 Department of Surgery and Cancer, Imperial College London, London W12 0HS, UK; nagy.habib@imperial.ac.uk (N.H.); skyntuh@gmail.com (K.W.H.); 3 Department of Surgery & Hepatitis Research Center, National Taiwan University Hospital, Taipei 100, Taiwan; 4 Centre of Mini-invasive Interventional Oncology, National Taiwan University Hospital, Taipei 100, Taiwan; 5 Graduate Institute of Clinical Medicine, College of Medicine, National Taiwan University, Taipei 100, Taiwan; 6 General, Emergency and Robotic Surgery Unit, San Francesco Hospital, 08100 Nuoro NU, Italy; mauropodda@ymail.com

**Keywords:** hepatocellular, carcinoma, immunomodulation, radiofrequency, checkpoint inhibitors

## Abstract

A majority of hepatocellular carcinoma (HCC) develops in the setting of persistent chronic inflammation as immunological mechanisms have been shown to play a vital role in the initiation, growth and progression of tumours. The index review has been intended to highlight ongoing immunological changes in the hepatic parenchyma responsible for the genesis and progression of HCC. The in-situ vaccine effect of radiofrequency (RF) is through generation tumour-associated antigens (TAAs), following necrosis and apoptosis of tumour cells, which not only re-activates the antitumour immune response but can also act in synergism with checkpoint inhibitors to generate a superlative effect with intent to treat primary cancer and distant metastasis. An improved understanding of oncogenic responses of immune cells and their integration into signaling pathways of the tumour microenvironment will help in modulating the antitumour immune response. Finally, we analyzed contemporary literature and summarised the recent advances made in the field of targeted immunotherapy involving checkpoint inhibitors along with RF application with the intent to reinstate antitumour immunity and outline future directives in very early and early stages of HCC.

## 1. Introduction

Hepatocellular carcinoma (HCC) is the most common primary liver malignancy; according to GLOBOCAN 2018 database, the figure of estimated annual incidence is approximately 841,000 and mortality of 782,000, labeling the entity as the sixth most commonly encountered and fourth most lethal cancer of the globe [1]. The HCC begins over the background of chronic inflammation of the liver, which occurs owing to several attributes, notably hepatitis B virus (HBV) and hepatitis C virus (HCV) infections and non-alcoholic steatohepatitis leading to cirrhosis [2,3,4].

Despite the recent advancement in the management of HCC, it continues to remain a significant burden on global health owing to late presentation, higher recurrence and metastasis. The disease prognosis is highly related to its stage at presentation; in an early stage with localised disease, hepatic resection or transplantation can be an option; a five-year survival is 40–70%; however, patients with advanced disease that is not amenable to locoregional therapy have a median survival rate between 3–11 months [5,6,7].

HCCs are classical archetypes of inflammation-associated malignancy as these tumours are arising in the context of hepatic inflammation and the resultant fibrosis. The risk factors of HCC lead to a chronic inflammatory state and proclivities of proinflammatory cells present within the tumour microenvironment engender carcinogenesis and dysregulated growth [8,9,10]; studies have outlined that continued expression of cytokines and recruitment of immune cells on the background of chronic inflammatory state cause DNA damage leading to genetic mutations and neoplastic transformation [11,12,13].

Typically, the host immune system perceives and eliminates any aberrant changes; however, that is not always the case and the inflammatory state of liver brings significant alteration in the tumour microenvironment to dodge the immune surveillance, secondary to changes in molecular and cellular pathways necessitated in antigen processing, presentation and degradation of HCC cells [14,15,16]. These changes bring a paradigm shift in the immune response from antitumour to the state of tumour tolerance, leading to genesis and progress of HCC [17,18,19]. Hence, there is a great deal of interest in understanding the immunopathogenesis of HCC in order to enhance the antitumour immune response or inhibit the suppressive effect of immunity as a potential source of immunotherapy. The present review has aimed to discern the main attributes of the very intricate and heterogeneous landscape of HCC, pivoting on the dynamic interplay between malignant and immune cells within the tumour microenvironment. On account of the fact that specific immune compositions may extend tumour growth, an improved understanding of the functioning of immune cells and better knowledge of diverse mechanisms of immune evasion will help in formulating various therapeutic approaches to modulate antitumour immune response in the management of very early and early-stage HCC tumours.

## 2. Immune Response and Tolerance in the Hepatic Parenchyma

The hepatic parenchyma performs multitudes of responsibilities such as removal of environmental or bacterial agents from the alimentary tract, elimination of bloodborne pathogens, and metabolizing and excreting various toxic substances. Concurrently, hepatic parenchyma has been exposed to loads of antigens, causing an enormous amount of immune response, which can cause collateral damage to the normal liver tissue. However, it gets countervailed through the intrinsic mechanism of hepatic parenchyma to tolerate the immune response [20,21,22].

The hepatic innate immune system, Kupffer cells, and liver sinusoidal endothelial cells (LSECs) play an important role in the induction of local immunosuppression [23]. The immune response includes differentiation of T cells into memory-like T-cells and generation effector T-cells following reactivation by antigen-presenting cells (APCs) as dendritic cells (DCs) and through activation of cytotoxic T--cells; however, in the situation of chronic inflammation and ongoing exposure of antigens induce tolerogenic hepatic priming [24,25]. The priming of hepatic parenchyma manifests with defective antigen processing by LSECs, undermines antigen-specific immune surveillance and declines in expression of co-stimulatory molecules B7-1 (CD80) & B7-2 (CD86) on CD4+ T-cells and CD137 on CD8+ and NK (Natural Killer) cells [26,27].

The CD80 (B7-1) and CD86 (B7-2) molecules present on different types of APCs play essential roles in the signaling of immune checkpoint pathways B7-CD28/CTLA-4. The interactions of B7 with the CD28 receptor on T-cells relay co-stimulatory signals to antigen-primed T-cells or may incur co-inhibitory signals following binding with the inhibitory checkpoint receptor, cytotoxic T-lymphocyte-associated protein 4 (CTLA-4) present on T-cells [20,28].

Further, the immunosuppressive role of CTLA-4 has been elucidated through the induction of immune tolerance in recipient following liver transplantation via expression of CTLA-4 molecule on Foxp3+CD25+CD4+ T-regulatory cells (Tregs), which indicates its applicability in the regulation of immune activity in liver transplantation [29,30].

Additionally, the PD-L1 molecules are present on hepatocytes, hepatic stellate cells (HSCs), LSECs, and Kupffer cells, which help in the genesis of immune tolerance through induction of T-cell dysfunction or apoptosis [31,32]. PD-L1/PD-L2/PD-1 (programmed death-ligand 1 or 2/programmed cell death 1 receptor) immune checkpoint pathway is involved in the inhibition of immune activity in the hepatic milieu, particularly in instances of chronic inflammation of the liver where the physiologic expression of PD-L1, along with PD-L2 and PD-1, get enhanced [28,33].

**Figure 1:**
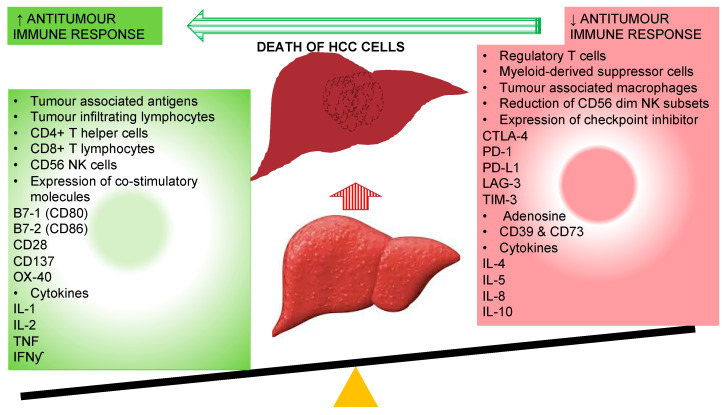
Immunological alteration associated with improved antitumour immune response with the resolution of hepatocellular carcinoma cells. CD: cluster of differentiation; CTLA-4: cytotoxic T-lymphocyte-associated protein-4; IFNγ: interferon-gamma; IL: interleukin; LAG-3: lymphocyte-activation gene 3; PD-1: programmed cell death-1; PD-L1: programmed cell death-ligand1; NK: natural killer cells; TIM-3: T-cell immunoglobulin mucin-3.

The main purpose of these tolerogenic responses is protection through avoidance of unnecessary inflammatory response against harmless antigens; however, they become inimical in a situation of chronic inflammation where immune tolerance to tumor-associated antigens (TAAs) contributes to onset and progression of HCC (Figure 1).

## 3. Tumour Microenvironment and Immunosuppression in HCC

The tumour microenvironment incites a multitude of changes for the genesis of HCC, involving immune cells and cytokines to elude the antitumour immune surveillance. Contemporary literature uncovered varied mechanisms of immune suppression and crosstalk between tumour cells, immune cells and microenvironment in modulating the process of liver fibrosis, hepatocarcinogenesis, epithelial-mesenchymal transition (EMT), tumour invasion, and distant spread [34,35,36]. Below we have outlined mechanisms employed to alter the antitumour immune response during the onset of HCC.

**Figure 2:**
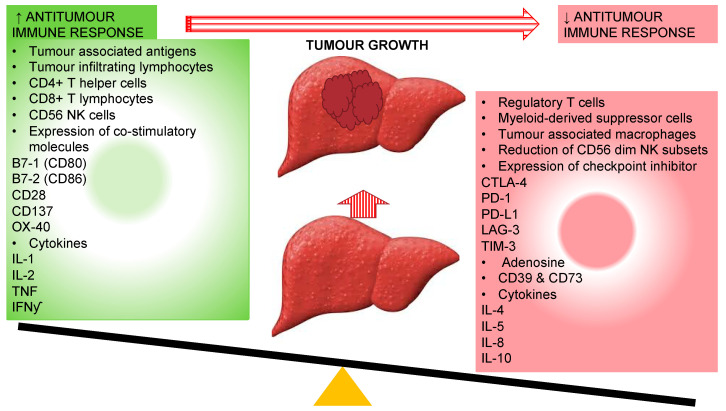
Different immune responses associated with genesis and progression of hepatocellular carcinoma owing to the reduction in antitumour immune response. CD: Cluster of differentiation; CTLA-4: cytotoxic T-lymphocyte-associated protein-4; IFNγ: interferon-gamma; IL: interleukin; LAG-3: lymphocyte-activation gene 3; PD-1: programmed cell death-1; PD-L1: programmed cell death-ligand1; NK: Natural Killer cells; TIM-3: T-cell immunoglobulin mucin-3.

The interactive immunosuppressive microenvironment interferes in detection of tumour antigen by DCs via downregulation of tumour associated antigen (TAA) and MHC molecules; secretion of inhibitory factors (IL-10, TGF-b, and VEGF) by tumour and tumour-associated macrophages to rivet suppressor cells in tumour microenvironment including Tregs cells, TAM, MDSC and immature DCs; VEGF mediated inhibition of differentiation and function of immune cells during hematopoiesis; inhibition of helper CD4+ T-cells; induction of MDSCs and Tregs secondary to secretion of immunosuppressive cytokines and release indoleamine 2,3-dioxygenase (IDO); inhibition of effector cells through Tregs cells, TAM, and MDSC via production of arginase, ROS, and suppressive cytokines IL-2 and TGF-b; reduced co-stimulatory molecule expression and activation of inhibitory receptors (CTLA-4 and PD-1) through its ligands, vitiating release of inflammatory cytokines IL-2, INF-y, TNF and cytotoxic chemicals perforin and granzyme by CD8+ T-cells [37,38,39,40]. On this account, it has been envisaged that the reinstatement of the antitumour immune response could have the potential of improving survival and decreasing recurrences in HCC (Figure 2).

## 4. HCC Tumour Microenvironment and Changes in Cytokines Milieu

Despite of enhanced levels of expression of neoantigens in HCC tissue and a reciprocal infiltration of CTLs in tumour tissue, the instigated antitumour immune response is flawed and inadequate [41]. The explicable explanations of diminished immune response include anergy of immune cells secondary to point mutation or insertion/deletion of β2 microglobulin present in MHC I molecules causing a defect in antigens presentation to T-cells; expression of inhibitory immune regulatory receptors, ligands and cytokines such as IL-10 and TGF-b. The immune checkpoints are inhibitory immune regulatory molecules, which include PD-1, PD-L1, CTLA-4, TIM3, lymphocyte-activating gene 3 protein and other lymphocyte attenuators. CTLA-4 expression on Tregs is associated with a decline in the production of granzyme B from CTLs, whilst its expression on CD14+ DCs relates with IL-10 and IDO mediated inhibition of T-cell proliferation with the induction of apoptosis. In addition, lowering of antitumour immunity is mediated through cells such as Tregs, MDSC and TAM involved in generating immune tolerance and reduction in hepatic parenchyma infiltrating CD56 dim NK, effector T-cells and CTLs [42,43,44].

To achieve optimal antitumor effects, CTLs must not only migrate to the tumour, but also be competent in functioning to induce lysis of tumour cells. In contrast to the earlier observation that increased density of lymphocytes within the HCC microenvironment is a surrogate marker of good prognosis following hepatic resection and transplantation [45]. Recent studies have highlighted that despite being the presence of adequate density of lymphocytes within HCC tissue, the antitumour immune responses are less than apposite owing to the diminution in the capacity for T-cells to proliferate, to release cytokine and inability to lyse tumour cells [46,47]. The abnormal set of behaviour can be explained by virtue of reduced CTLs activity secondary to suppressive nature of HCC tumour microenvironment and failing to release IFNγ upon stimulation of tumour-infiltrating CD8+ T-cells in contrast to peripheral CD8+ T-cells [48], although the installation of CTLs following isolation and ex-vivo cultivation has exhibited optimum antitumour specific activity [49]. Further research has been made to understand the clinical implication of these changes in immunological parameters in terms of survival. A recent study has outlined poor overall survival in HCC patients with increased levels of Tregs and reduced intra-tumoural and peripheral CD8+ T-cells [50]. Finkelmeier et al. (2016) investigated the prognostic value of soluble PD-L1 with HCC and concluded that higher values are associated with dismal outcomes [51]. Similarly, an increase in levels of immunosuppressive cytokines (IL-4, IL-5, IL-8 and IL-10) or diminution of stimulating cytokines (IL-1, TNF-a, TFN-y) are also considered as a marker of poor prognostic value [52,53,54].

Even though the pathways entailed in HCC associated immune tolerance have not been completely elucidated, contemporary research has outlined the potential of targeted therapy in the reinstatement of antitumour immunity through the interaction of receptors, ligands and instigating immune cells and cytokines level [55].

Tregs cells, one of the important constituents of the HCC tumour microenvironment, cognate with a reduction in density and functioning of CD8+ T-cells, hence a therapeutic measure targeting Tregs would not only help in establishing of antitumour immune responses but can also improve survival [56,57]. Studies have outlined a significant decline in levels of intra-tumoural and circulating Tregs cells following radiofrequency ablation or radiofrequency based resection of HCC tumour and an associated improvement in survival [58].

## 5. HCC Tumour Microenvironment Immunomodulation

### 5.1. HCC Tumour Microenvironment Immunomodulation through Radiofrequency Application

**Figure 3:**
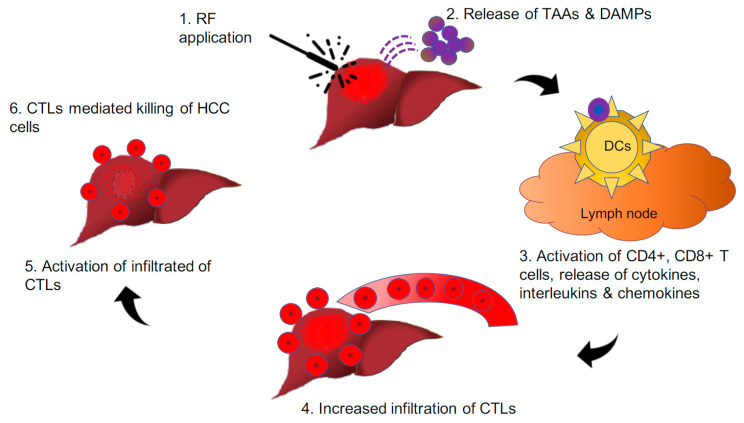
Pictorial depiction of the hypothesis that “in-situ vaccine effect” through radiofrequency induces the release of neoantigens and DAMPs from HCC nodules, leading to the reinstatement of the antitumour immune response.

Contemporary research has demonstrated that the application of radiofrequency (RF) over HCC nodules not only kills the tumour cells but also releases an abundance of neoantigens and DAMPs, which, in turn, incites CD8+ T-cell infiltration. The CD8+ T-cells then recognise such antigen-producing tumour cells alongside metastatic cells. Such immune-mediated response to locoregional therapy over the tumour nodule was first described in relation to radiation and is known as the “abscopal effect” [59]. The mechanism behind the abscopal effect has not been completely elucidated; a multitude of studies have outlined the potential of combining neoantigen generating locoregional therapy with immunotherapy could further enhance boost such effect. The cellular stress following tumour irradiation generates tumour-associated antigens (TAAs) secondary to necrosis and apoptosis of tumour cells and debris similar to vaccine effect with intent to treat or prevent the development of malignancy [60] (Figure 3). Such effects are observed in various solid tumours, including melanoma, renal cell carcinoma, hepatocellular carcinoma, to name a few. Further, radiofrequency waves are similar to radiation, which can elicit a tumour-specific in situ vaccine effect, resulting in a systemic response. Hence, the application of radiofrequency energy over the HCC tumour facilitates the release of DAMPs with subsequent increase in peripheral and tumour infiltrating CD4+, CTLs and NK cells, which shift the scale of balance towards the antitumour immune response rather than cancer progression [61,62].

### 5.2. HCC Tumour Microenvironment Immunomodulation through Immunotherapy

As mentioned earlier and demonstrated in various studies, a mere increase in the levels of immune cells is not adequate to generate sufficient immune response owing to anergic T-cells. Hence, immunotherapy targeting co-stimulatory and inhibitory (checkpoint) receptors can reestablish the T-cell priming and effector functioning back to normal [63]. A targeted approach to modulate the activity of T-cells includes antagonism of checkpoint inhibitors. Of the various molecules involved in the immune checkpoint, PD-1, PD-L1 and CTLA-4 have been shown to play important roles in suppression of T-cell activation by malignant cells [64,65]. On continuance, the development of monoclonal antibody targeting these molecules has been found to be highly efficacious in cancer with high immunogenicity, such as malignant melanoma. The last decade has witnessed significant progress in the understanding of the immune system and led to the development of immune checkpoints blockades such as anti-CTLA-4, anti-PD-1 and anti-PD-L1, which has shown the potential to bring a paradigm shift in management and prognosis of several cancers, including liver cancer [55,65].

The expression of CTLA-4 is increased following the activation of T-cells. The interaction between CTLA-4 and its ligand not only inhibits activity but also inflict anergy to T-cells. The anti-CTLA-4 monoclonal antibodies (tremelimumab and ipilimumab) have demonstrated their ability to deplete Tregs cells and reverse exhaustion of T-cells with intent to reinstituting the antitumour immune response. An anti-CTLA-4 monoclonal antibody (tremelimumab) has received FDA approval in 2011 for the treatment of malignant melanoma [66,67,68].

Similarly, PD-1 and PD-L1 get upregulated following the activation of T-cells and the coupling of PD-1 with PD-L1 leads to inactivity of T-cells and NK cells functioning. The notable anti-PD-1 (nivolumab and pembrolizumab) and anti-PD-L1 (atezolimumab and avelumab) have further expanded the applicability of checkpoint inhibitors to various other cancers, including HCC. Presently, several phase III trials are going on, and the scientific community is eagerly waiting to peruse their outcomes and explore future applicability [64,69] (Table 1).

A phase II, non-controlled trial evaluated tremelimumab, a humanised IgG2 monoclonal antibody against CTLA-4 in advanced HCC patients not eligible for surgery or locoregional therapy. A total of 21 patients with advanced HCC with chronic HCV infection and Child–Pugh scores A (57.1%) or B (42.9%) were enrolled. Each patient received 15 mg/kg of tremelimumab every 90 days as a single agent therapy. A partial response was observed in 17.6% with a disease control rate of 76.4% and a median overall survival of 8.2 months. Approximately 45% had transient grade 3 transaminase toxicity following initial dosing of tremelimumab dose, although it did not require systemic steroids. A progressive decline in viral load was observed most of the patients, but a complete viral response was reported in just 3 patients. The analysis of results indicates a dual effect of tremelimumab in terms of antitumour and antiviral activity, suggesting that immune checkpoint treatment can be particularly beneficial in patients with a viral etiology such as HBV or HCV-related HCC [70].

A phase I/II trial studied the safety and preliminary activity of anti-PD-1 antibody nivolumab (humanised IgG4 monoclonal antibody) in 262 advanced HCC patients with or without HBV or HCV infection received 0.1–10 mg/kg of nivolumab once every 2 weeks (dose-escalating cohort) or at a dose level of 3 mg/kg once every 2 weeks (expansion cohort). The trial demonstrated a manageable safety profile with a promising efficacy (dose-escalation cohort: response rate of 15%, median survival period of 15 months; expansion cohort: response rate of 20%, duration of response 9.9 months [71]. Further phase III randomised trial (NCT02576509) has compared nivolumab against sorafenib for unresectable HCC as a first-line treatment but did not outline any significant difference in terms of overall survival (HR, 0.85; 95% CI, 0.72–1.02; *p* = 0.0752); however, further details of analysis by study group are still awaited [72]. Another ongoing trial NCT03383458 is evaluating the efficacy of nivolumab as adjuvant therapy following surgical resection or ablation for HCC tumours [73].

Another phase II trial (KEYNOTE-224, NCT02702414) with anti-PD-1pembrolizumab reported overall response rate (18%) and median survival of 12.9 months in advanced HCC following failed sorafenib treatment. In light of these promising results, the US FDA granted approval to nivolumab and pembrolizumab for treating HCC in patients who had received prior sorafenib, while many other immune checkpoint inhibitors are under evaluation to determine their applicability for treatment in HCC [74] (Table 1).

Studies have advocated that combining anti-CTLA-4 with anti-PD-1/PD-L1 could produce a superlative approach in reestablishing a competent immunity through the mitigation of immunosuppression signals. The interaction of CTLA-4 on T-cells with B7 ligands expressed on DCs or APCs in lymph node limits number and activity T-cells, whereas binding of PD-1 expressed on the activated CTLs to its ligand PD-L1 on tumour cells or TAM, brings inactivity of T-cells [75,76]. Hence, the rationale of combining includes induction of T-cells proliferation through inhibition of CTLA-4 and enhance CTLs activity through PD-1 inhibition.

A phase I/II trial assessed the combination of anti-PD-L1 antibody (durvalumab) and anti-CTLA-4 antibody (tremelimumab) in 40 patients with advanced HCC and demonstrated a response rate of 25%; highlighting the benefit of combined approach over monotherapy with tolerable toxicity profile. Presently, phase III trial (NCT03298451) is evaluating the efficacy of various regimens, including durvalumab monotherapy with two regimens of durvalumab and tremelimumab combination and sorafenib monotherapy. Another ongoing trial (NCT01658878) is assessing the efficacy of combination nivolumab with ipilimumab in contrast to nivolumab alone [74,77].

### 5.3. HCC Tumour Microenvironment Immunomodulation through Combined Approach

Contemporary research has demonstrated locoregional therapy, particularly radiofrequency (RF) based ablation of HCC nodules, not only kills the tumour cells but also release an abundance of neoantigens and DAMPs and induce CD8+ T-cell infiltration. According to meta-analysis performed by Ding et al., increased density of tumour infiltrating lymphocytes (TILs) have been significantly associated with improved survival [78]. Additionally, studies have outlined positive immunomodulatory change following the application of RF in terms of Tregs, CD8+ T-cells, TGF-β, IFNγ, IL-10, IL-17, respectively [79,80,81,82].

**Figure 4:**
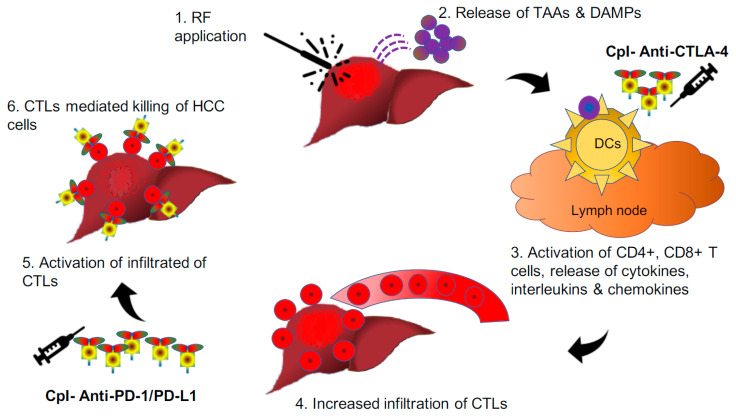
Pictorial depiction of radiofrequency-induced release of neoantigens and DAMPs from HCC nodules whereupon activation of CD4+ and intratumoural infiltration CD8+ T-cells. The activity of these cells gets further augmented with the introduction of checkpoint inhibitors. CD: cluster of differentiation; CpI: checkpoint inhibitors; CTLA-4: cytotoxic T-lymphocyte-associated protein-4; DAMPs: damage-associated molecular patterns; PD-1: programmed cell death-1; PD-L1: programmed cell death-ligand1; RF: radiofrequency.

Further, Tumeh et al. (2014) demonstrated that better tumour response following introduction pembrolizumab in a situation of higher expression of PD-1/PD-L1 on CD8+ T-cells at the margin of melanoma tumours. In addition, observation of significant tumour regression was discerned in association with an increase in CD8+ T-cells from baseline to post-treatment biopsy, specifically at the tumour center and invasive margin. Hence, both baseline and post-treatment CD8+ T-cells may act as important biomarkers in envisaging the tumour response to checkpoint inhibitors [83]. The combined approach involves radiofrequency ablation to generate neoantigens and influx of CD8+ T-cells, along with checkpoint inhibitors to activate these CD8+ T-cells to invigorate an antitumour immune response against HCC cells. Here, RF-induced cellular stress generates tumour-associated antigens (TAAs) through necrosis and apoptosis, which act as vaccines to activate the antitumour immune response, which gets boosted with the simultaneous introduction of checkpoint inhibitors with intent to treat or prevent the development of malignancy and distant metastasis (Figure 4). The same principle has formed the basis of a recent trial done by Duffy et al. (2017), where they evaluated 19 patients of advanced HCC to understand the clinical response of combining ablation with anti-CTLA-4 (tremelimumab). Five (26.3%) of nineteen had a partial response (95% confidence interval, 9.1–51.2%); 12 out of 14 HCC patients also marked quantifiable reduction in HCV viral load following treatment [84]. The plausible explanation of the observed decline in viral load could be because of the simultaneous return of immune response against these hepatotropic viruses too. Although the observed findings are intriguing and require further studies to envisage future applicability.

Immune checkpoint inhibitors are proved to be beneficial in the treatment of advanced HCC; however, the world of immunomodulation is still not fully explored, particularly in very early and early stages of HCC. Moreover, the identification of predictive markers is of the utmost importance to determine a subgroup of HCC patients who are most likely to be benefitted from checkpoint inhibitors. Research exploring predictive markers for checkpoint treatment response has pointed towards mutational burden, PD-L1 expression, expression of immune regulatory molecules and epithelial-to-mesenchymal transition (EMT) [85,86]. Shrestha et al. (2018) [87] reported that HCC patients with higher mutation burden have significantly poor overall survival and progression-free survival than those with a lower mutation burden; however, a study by Mauriello et al. [88] highlighted that high mutation does not correlate with a decline in survival in the absence of immunotherapy. On the contrary, multiple studies have shown that a high mutation burden associated with a better response to checkpoint inhibitors in melanoma patients. The mechanism is not fully understood, but an increased number of neoantigens (potential tumor-specific T-cell targets) generated by a high mutation burden is thought to bring an enhanced response from checkpoint inhibitors. Similarly, PD-L1 expression in HCC has been reported as a predictive biomarker for poor prognosis and can also utilised as an important tool to predict the response to anti-PD-1 antibodies; however, identifying other immune biomarkers could play an important role to further improve patient outcome as PD-L1 levels fluctuate along the course of the disease and only expressed in 30% of HCC tumours [89]. Higher levels of PD-L1 induce EMT in HCC patients with an increase in invasion and metastasis of malignant cells [87,90]. Thus, HCC patients with EMT phenotype are more likely to respond to PD-1/PD-L1 targeted immunotherapy.

## 6. Future Perspectives and Conclusions

Despite advancement in chemotherapy, the observed outcomes in advance HCC tumours are dismal owing to reduced immune recognition of cancer cells and the development of an immunosuppressive microenvironment, rendering the immune system unable to mount an effective antitumour response. The present review has outlined the tumour microenvironment in HCC and summarised the applicability of checkpoint inhibitors and their potential benefits in terms of partial response, reduction in viral load and improved survival in advanced HCC. The same principle could be applicable in very early stages of HCC, where radiofrequency ablation or radiofrequency based resection in early stages to generate neoantigens to reinstate antitumor immunity, which could be boosted with checkpoint inhibitors. The mechanisms behind the diverse clinical responses to mono or a combined regimen of checkpoint inhibitors in advanced HCC patients are insufficiently elucidated. A focus on functional evaluations in the context of patient immunity for novel regimens alongside clinical testing may shed some light on the most effective combinations and the best strategies to reduce adverse effects. However, because of the complexity of the antitumour immune response and the observed heterogeneity, it is improbable to predict wide-ranging clinical benefits without using a wide set of biomarkers. Biomarker development is an area of intense study and remains a considerable clinical challenge. A few notable markers for checkpoint treatment response include the degree of mutational burden, PD-L1 expression, expression of immune regulatory molecules and EMT phenotype; however, further studies are warranted to develop better strategies with checkpoint inhibitors in HCC along with biomarkers in very early and early stages.

## Figures and Tables

**Table 1:** Clinical Trials involving various immune checkpoint inhibitors in hepatocellular carcinoma.

Name	Study	Detail	Phase	Sample	Primary Endpoint	Status
CTLA-4	NCT01853618	Tremelimumab with ablation	II	32	PR—26% Median TTP—7.4 mon Median OS—12.3 mon	Completed
	NCT02519348	Tremelimumab with Durvalumab	II	144	Safety and tolerability	Ongoing
PD-1	NCT01658878	Nivolumab vs. Placebo	I/II	262	OR—20% DOR—9.9 mo	Completed
	NCT02576509	Nivolumab vs. Sorafenib	III	726	Overall survival	Completed (Results awaited)
	NCT02702414	Pembrolizumab vs. Sorafenib	II	104	OS—26% Median OS—12.9 mon	Completed
	NCT02702401	Pembrolizumab vs. Placebo	III	408	Progression-free survival, Overall survival	Ongoing
	NCT03062358	Pembrolizumab vs. Placebo	III	330	Overall survival	Ongoing
	NCT03383458	Nivolumab vs. Placebo	III	530	Recurrence-free survival	Ongoing
	NCT02512773	Tislelizumab vs. Sorafenib	III	660	Overall survival	Ongoing
PD-L1	NCT03298451	Durvalumab vs. Durvalumab + Tremelimumab (regimen 1) vs. Durvalumab + Tremelimumab (regimen 2) vs. Sorafenib	III	1200	Overall survival	Ongoing
	NCT03434379	Atezolizumab + Bevacizumab vs. Sorafenib	III	480	Overall survival	Ongoing

## Abbreviations

CTLA-4: cytotoxic T-lymphocyte-associated protein-4; PD-1: programmed cell death-1; PD-L1: programmed cell death-ligand1; OS: overall survival; DOR: duration of response; PR: partial response; mon: month.
